# Case Report: A report of two cases and literature review: primary pulmonary Ewing sarcoma presenting with hemoptysis and back pain

**DOI:** 10.3389/fonc.2026.1880366

**Published:** 2026-08-21

**Authors:** Yunlong Guo, Jiaqi Wang, Guangxin Zhang, Chengyan Jin

**Affiliations:** Department of Thoracic Surgery, Second Hospital of Jilin University, Changchun, China

**Keywords:** back pain, Ewing sarcoma (EWS), hemoptysis, primary pulmonary Ewing sarcoma (PES), pro-gastrin-releasing peptide (ProGRP), small cell lung cancer (SCLC)

## Abstract

**Background:**

Primary Pulmonary Ewing Sarcoma (PES) is an extremely rare and lethal subtype of Ewing sarcoma (EWS) arising from the lungs, and predominantly affects young adults. The available literature is limited. Typically, affected individuals exhibit respiratory distress, cough, and chest pain; hemoptysis or back pain as the chief presenting symptom is highly uncommon.

**Case presentation:**

The paper presents two cases of PES in which hemoptysis or back pain was the main clinical symptom. The first patient was a 42-year-old female presenting with hemoptysis, and the second was a 65-year-old female admitted for two months of persistent back pain. Neither patient had a history of smoking, pulmonary disease, family malignancy, or known genetic predisposition to cancer. Chest computed tomography (CT) scans revealed consistent findings in both patients: irregular, mass-like soft-tissue opacities adjacent to the pulmonary hila. Based on the initial clinical manifestations and CT findings, bronchial adenocarcinoma or squamous cell carcinoma was initially suspected. Both cases underwent surgical intervention. However, small cell lung cancer (SCLC) could not be excluded based on chest CT findings, preoperative lung tumor marker levels, and intraoperative frozen-section pathological consultation results. Immunohistochemistry (IHC) and molecular analysis were used to arrive at the final diagnosis. Case 1 showed strong diffuse positive staining for CD99, FLI-1, and NKX2.2, along with confirmed EWSR1 gene rearrangement. The diagnosis of PES in Case 2 was confirmed by the highly specific dual positive expression of CD99 and NKX2.2, despite the absence of molecular testing.

**Conclusion:**

For middle-aged and young non-smoking patients with hemoptysis and no other lung cancer risk factors, as well as elderly patients presenting with predominant back pain, PES should be included in the differential diagnosis even in the presence of elevated preoperative pro-gastrin-releasing peptide (ProGRP) levels and intraoperative pathological findings suggestive of SCLC. Supplemental IHC and molecular analyses are essential to ensure an accurate definitive diagnosis and guide optimal therapeutic strategies.

## 1 Introduction

Ewing sarcoma (EWS) is a highly aggressive malignant tumor that predominantly affects children and adolescents (1), with rare occurrences in adults. EWS typically arises from the skeletal system, while approximately 15% of cases occur in extraskeletal sites (2), including the lungs, liver, and adrenal glands (3). Among these extraskeletal sites, the chest is the most common extraosseous site of EWS in children, although it is uncommon in adults (4). Adult PES is extremely rare and typically presents with dyspnea, cough and thoracic pain, which is easily misdiagnosed as lung cancer. Hemoptysis or back pain are rare primary symptoms of PES. A 14-year retrospective analysis of 50 PES cases reported only three cases with hemoptysis (5–8) and one case with back pain (9).

The current report presents two cases of PES in non-smoking female patients. One middle-aged patient presented with hemoptysis, while one elderly patient presented with predominant back pain. Both cases mimicked SCLC in preoperative and intraoperative manifestations and were ultimately confirmed as PES via IHC and molecular testing. Based on these two diagnostically challenging cases, this study aims to improve the diagnostic proficiency of clinicians and pathologists for this rare disease and provide evidence-based references for the evaluation of similar clinical cases.

## 2 Case presentation

### 2.1 Case report 1

**Figure 1:**
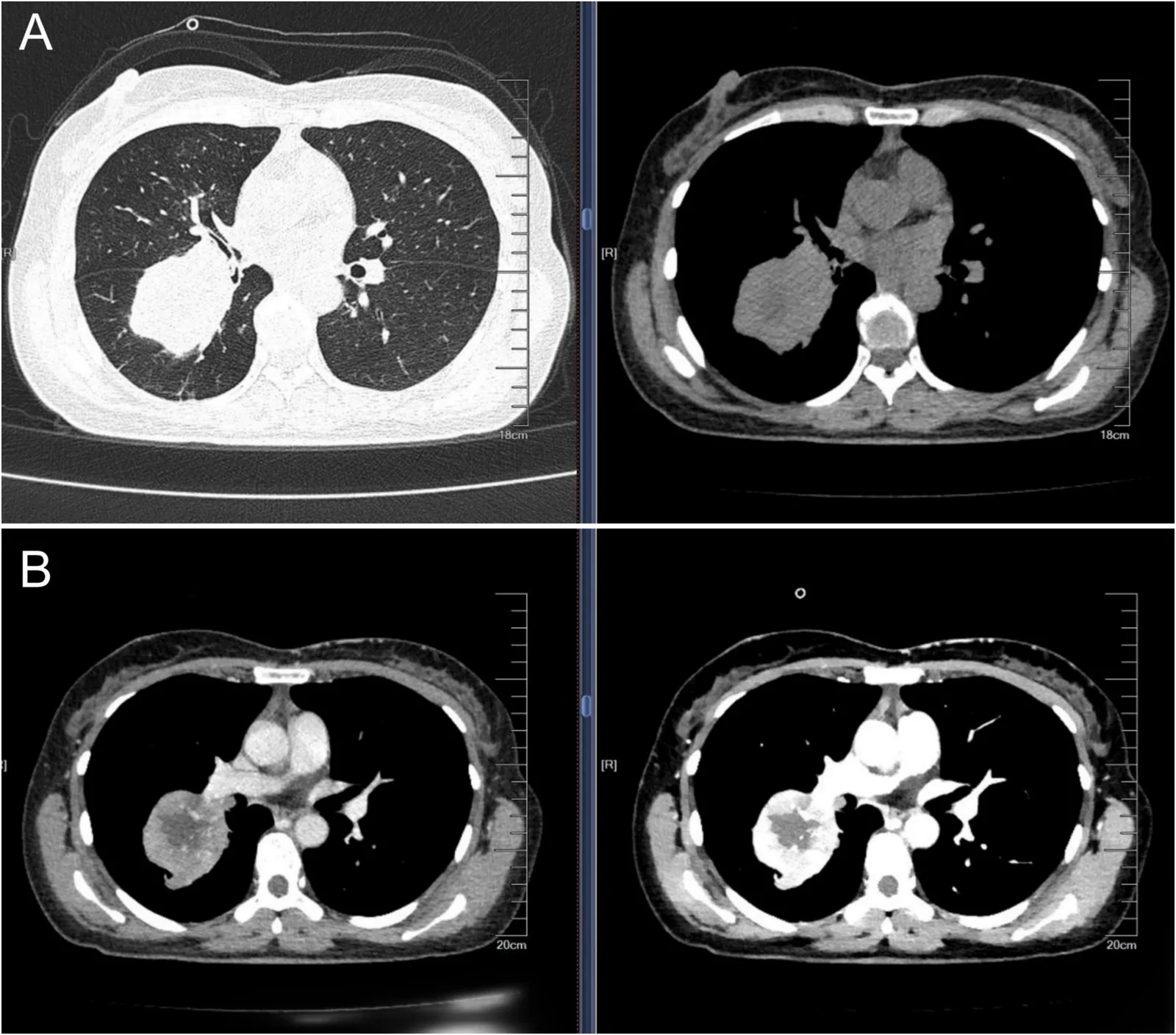
Preoperative axial chest CT series. **(A)** Non‑contrast axial chest CT (lung window and mediastinal window) showing an approximately 5.5 cm × 4.8 cm heterogeneous soft‑tissue mass with ill‑defined borders adjacent to the right pulmonary hilum. **(B)** Contrast‑enhanced axial chest CT (mediastinal window) demonstrating peripheral enhancement and central necrosis within the lesion.

The patient was a 42-year-old female. She was admitted following two sudden episodes of hemoptysis, with an estimated volume of approximately 30 mL. The patient had no previous chronic lung disease and was a healthy non-smoker prior to this episode. Chest CT demonstrated an ill-defined irregular mass-like soft-tissue opacity adjacent to the right pulmonary hilum, measuring 5.5 cm x 4.8 cm. Contrast-enhanced CT imaging demonstrated that the lesion showed heterogeneous enhancement, including areas of necrosis (**Figure 1**), but the ribs and the pleura were not involved by the tumor. No additional masses or distant metastases were detected on cranial CT, total abdominal CT and whole-body bone scan. Given the high clinical suspicion of pulmonary malignancy, surgical intervention was performed.

**Figure 2:**
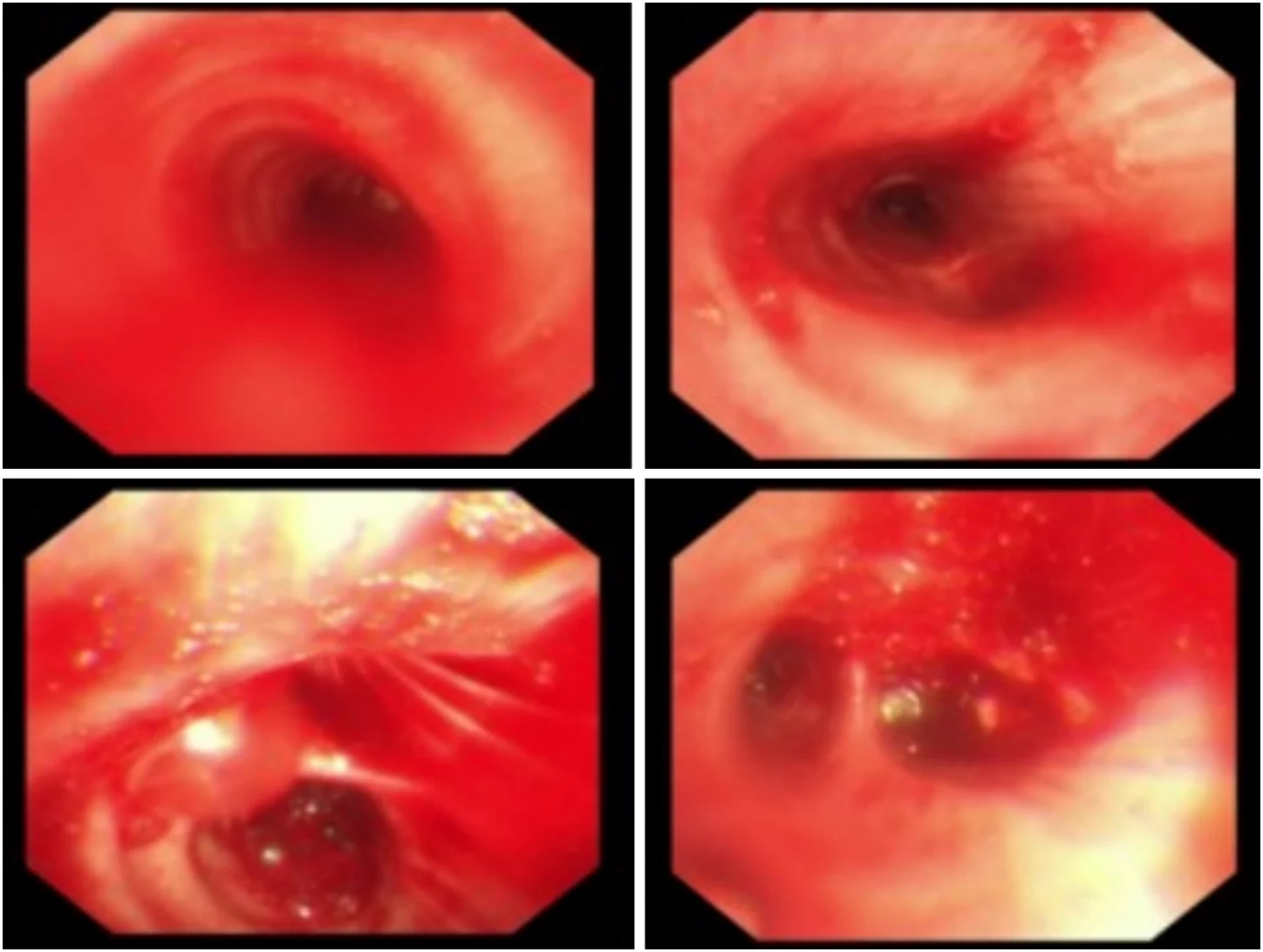
Bronchoscopy series. Bronchoscopic examination reveals extensive fresh bleeding in both main bronchi and in all lobar and segmental bronchi of both lungs. After clearing the bloody secretions, active bleeding remains evident at the orifice of the right lower lobe bronchus.

The patient experienced approximately 60 mL of bright red hemoptysis during the peri-admission and admission period. Hemostasis was administered with Pituitrin. Bronchoscopy was performed to identify the bleeding site and characterize the lesion (**Figure 2**). The examination revealed extensive fresh bleeding that obscured the visualization of the trachea, lobar bronchi and segmental bronchi, posing a life-threatening risk. Severe active bleeding was observed at the orifice of the right lower lobe bronchus. The intraoperative blood loss was about 100 ml, which precluded visualization of the right lower lobe bronchial orifice and biopsy collection. Hospitalization was accompanied by recurrent intermittent hemoptysis, with a cumulative volume greater than 100 ml and a hemoglobin concentration of 82 g/L(moderate anemia). Acute hemoptysis on the day of surgery prompted urgent re-evaluation. After detailed risk communication with the patient’s family and acquisition of informed consent, emergency surgery was performed.

**Figure 3:**
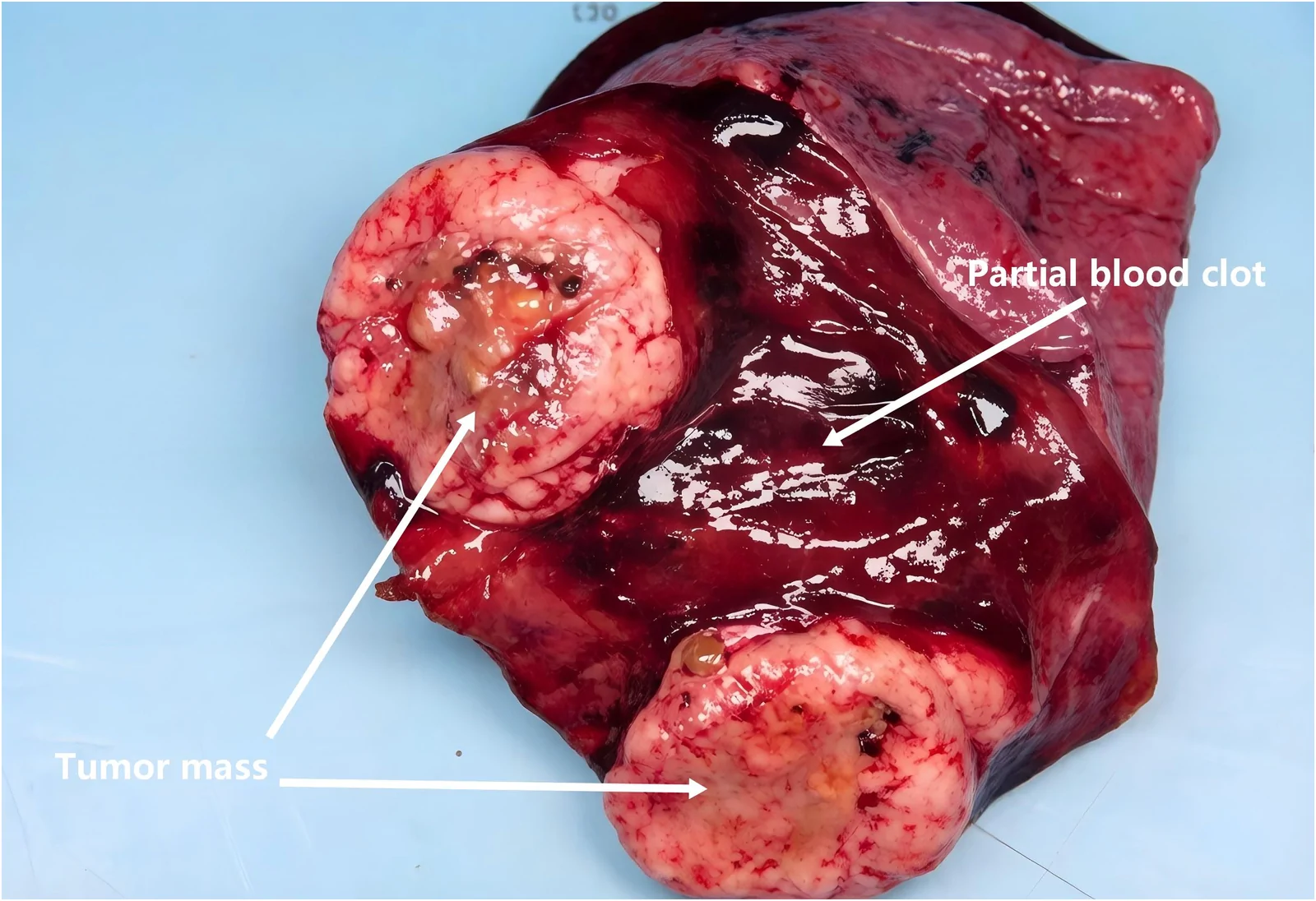
Gross specimen of the surgically resected right lower lobe. The left arrow indicates the tumor tissue within the right lower lobe, while the right arrow points to a portion of the blood clot.

**Figure 4:**
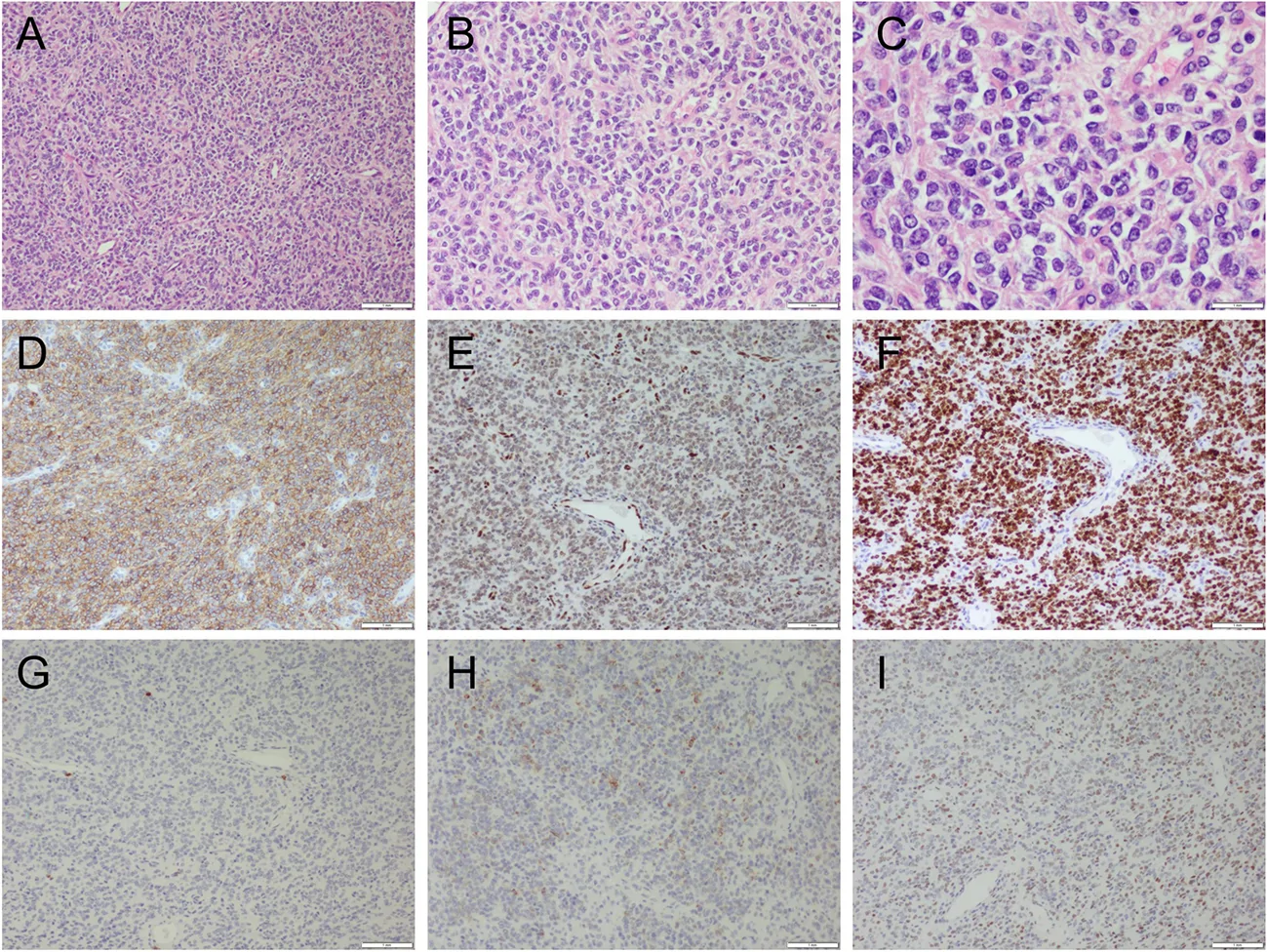
Histopathological series. **(A–C, E, H)** staining. Densely packed small blue round cells with occasional mitotic figures are seen in the post_resection specimen [**(A)** ×10 magnification; **(B)** ×20 magnification; **(C)** ×40 magnification). **(D–I)** Immunohistochemical staining. Panels **(D–F)** show diffuse positivity for CD99, FLI1, and NKX2.2, respectively. Panel **(G)** demonstrates scattered S_100 positivity. Panels **(H, I)** are focally positive for CK and TLE1, respectively.

Upon a posterolateral thoracotomy, a 6 cm round mass was identified in the right lower lobe, and right lower lobectomy was performed. Only congestion was observed in the medial segment of the right middle lobe. The excised tumor was pale-gray, soft, and pleural-adjacent, and had significant blood clots in the bronchial lumen (**Figure 3**). Frozen section findings during surgery revealed a neoplastic lesion (right lower lobe) with evident mitotic figures, which were suspected to be malignant; intraoperative pathology reported small round cells (**Figures 4A–C**) and could not rule out SCLC. The mediastinal lymph nodes were then dissected. With an intraoperative blood loss of 150 ml combined with preoperative anemia, the patient received transfusion of 1.5 units of red blood cell (RBC) suspension and 370 ml of plasma. The patient achieved uneventful postoperative recovery with routine anti-inflammatory, analgesic and hemostatic treatment. The postoperative hospital stay lasted 11 days. The pathological diagnosis after surgery was EWS (right lower lobe) and it was confirmed by morphology, IHC and a positive EWSR1 fluorescence *in situ* hybridization (FISH) result indicating gene breakage. The tumor measured 6 cm in diameter without spread through air spaces (STAS) or lymphovascular invasion. Pathological stage was pTNM: T3N1Mx. IHC findings were:CK (AE1/AE3) (partial +), CD99 (+), TLE1 (focal +), CD117 (focal weak +), FLI-1 (+), NKX2.2 (+), S-100 (scattered +); TTF-1, CD34, STAT6, HMB45, P40, WT1, ERG, Desmin, BCOR and CD56 were negative. The Ki-67 proliferation index was 30%.

Due to convenient local medical follow-up conditions, the patient was transferred to a local hospital after discharge to receive six alternating cycles of adjuvant VAC/IE chemotherapy regimen (vincristine, epirubicin, and cyclophosphamide alternating with ifosfamide and etoposide), with a three-week treatment interval per cycle. The exact plans were (1): VAC cycle: vincristine (2.0 mg/day, day1 and day8, intravenous administration), epirubicin (60 mg/day, day1-day3, intravenous infusion), and cyclophosphamide (1.2 g/day, day1, intravenous administration) (2); IE cycle: ifosfamide (2.0 g/day, day1-day5, intravenous infusion) and etoposide (120 mg/day, day1-day5, intravenous infusion). Follow-up data indicated that the patient has completed four alternating cycles of adjuvant chemotherapy to date. The patient remained in stable mental status with mild nausea, vomiting and hepatic dysfunction, and exhibited good treatment tolerance.

### 2.2 Case report 2

**Figure 5:**
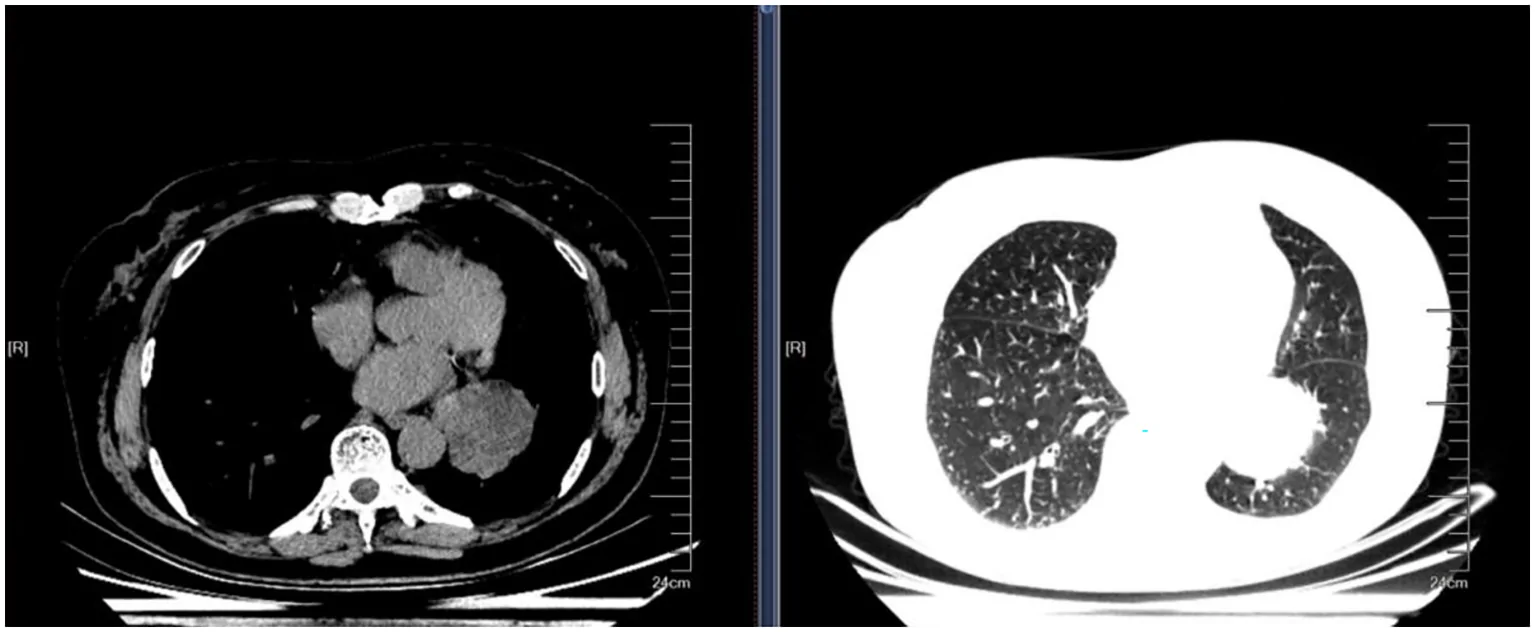
Postoperative histological series. **(A)** Gross photograph of the partially resected left lower lobe, showing a tumor mass (arrow) measuring approximately 5.3 cm in diameter. **(B)** Hematoxylin and eosin (H&E) staining demonstrates sheets of small blue round cells, consistent with malignant cytomorphology.

The patient was a 65-year-old non-smoking female with no prior pulmonary disease history, who presented with predominant back pain. A 5 cm irregular soft-tissue opacity was identified adjacent to the left pulmonary hilum on CT (**Figure 5**), with no evidence of rib destruction or pleural invasion. No other primary or metastatic lesions were found on systemic imaging workup, including cranial MRI, abdominal CT, and whole-body bone scan. Transbronchial aspiration cytology (TBAC) and biopsy of the B10 bronchial segment were performed. Microscopic examination demonstrated extremely compressed and hyperchromatic aggregates of cells with a pattern of invasive growth indicating malignancy. IHC findings: Rb (partial +); CK (AE1/AE3), TTF-1, P40, CgA, Syn, CD56, INSM1, LCA, NUT, and P53 all negative and a Ki67 index of 40%. The unusual IHC profile did not rule out SCLC. Bronchial lavage cytology also indicated possible SCLC. Surgical resection was performed due to insufficient biopsy specimens for definitive molecular testing and the patient’s family’s strong preference for surgery.

**Figure 6:**
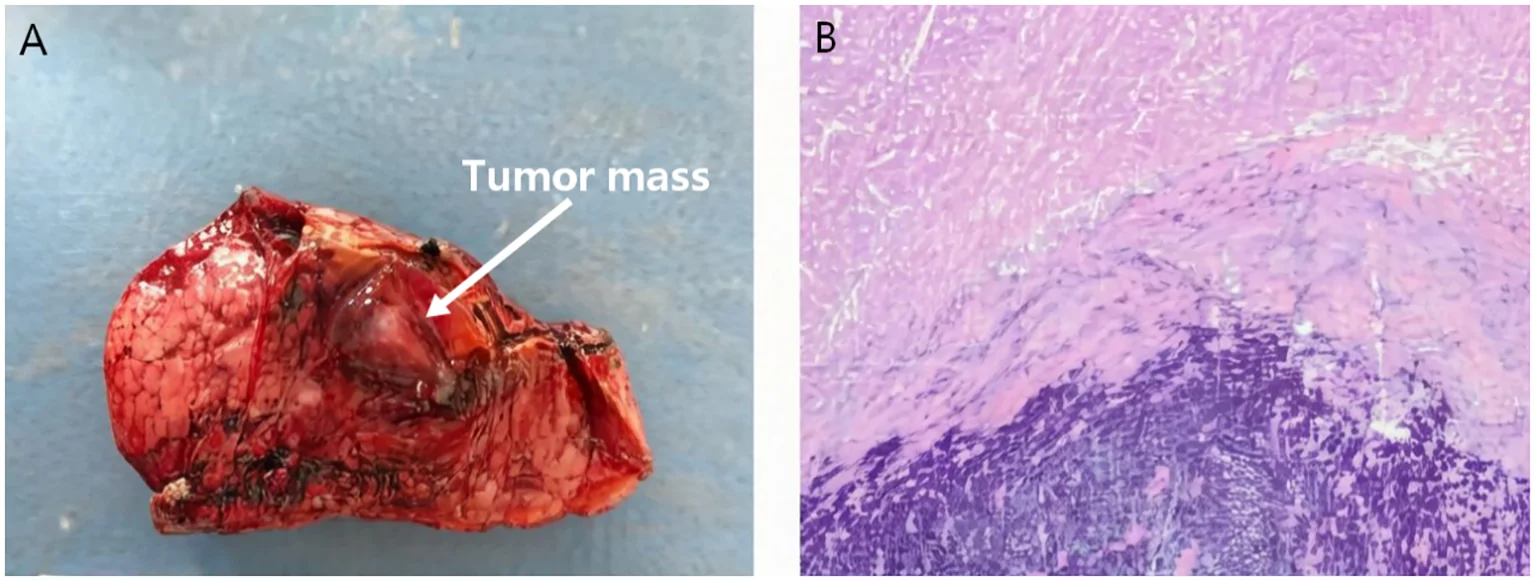
Preoperative axial chest CT series. Preoperative axial chest CT images (lung and mediastinal windows) demonstrate an approximately 5-cm, lobulated, heterogeneous soft-tissue mass adjacent to the left pulmonary hilum.

During the posterolateral thoracotomy, an intrapulmonary mass that measured 5 cm in its largest dimension was found in the left lower lobe. Because of the dense hilar adhesions, the wedge resection was done rather than the lobectomy (**Figure 6**). Given that intraoperative frozen section analysis could not exclude SCLC, and in accordance with oncological protocols for suspected SCLC based on tumor location, size, and radiological features, a mediastinal lymphadenectomy was performed. Postoperative pathology revealed undifferentiated round cell sarcoma consistent with EWS. The results of the IHC were: CD99 (+), NKX2.2 (+), Ki67 (40%), ERG (–), S-100 (–), FLI-1 (–), WT1 (–), and Desmin (–). Even though EWSR1 FISH could not be done because of limited material and financial resources, the double-positive staining of CD99 and NKX2.2 with the morphology alone was adequate to diagnose PES. The patient refused postoperative adjuvant chemotherapy and was eventually lost to follow-up.

## 3 Discussion

Ewing sarcoma family of tumors (ESFT) represents a wide range of neoplasms including classic skeletal EWS, extraosseous EWS, Askin tumors and peripheral Primitive Neuroectodermal Tumors (pPNETs) that have been associated due to the similarity of their histological and clinical characteristics (10). One of the diagnostic criteria of these tumors is the neuroectodermal origin, which is visible microscopically as a significant population of small round cells. The presence or absence of neural differentiation is the sole criterion for distinguishing EWS from pPNETs. Specifically, pPNETs are defined as tumors with advanced neural maturation, while EWS lacks such differentiated features (11). PES is a rare extraosseous variant of EWS and only about 50 cases have been reported globally (5). The clinical presentation is dominated by cough, and other commonly found symptoms include respiratory distress and thoracic discomfort. Of the 50 cases reported, hemoptysis was seen in only three patients: two male patients (aged 42 (6) and 31 (7)) and one female patient (aged 41 (8)). Back pain has only been reported in one case of PES (a 57-year-old woman (9)).

The hemoptysis in Case 1 could have been caused by the rich blood supply of the tumor or the invasion of vessels as shown in enhanced CT (**Figure 1B**). Literature shows that the occurrence of hemoptysis in EWS could be related to the highly invasive nature of the tumor or extensive necrosis (12). However, in lung cancer, hemoptysis is also commonly observed in squamous cell carcinoma and SCLC. Back pain, a hallmark symptom of lung cancer in elderly patients, initially misled the clinical diagnosis in Case 2. In contrast, the typical patient population for SCLC and squamous cell lung cancer includes older male smokers with a long history of smoking, which is not consistent with the picture of a middle-aged non-smoking female patient in this case. This complicates the diagnostic process.

In the radiological aspect, Case 1 had fairly smooth margins, which is more typical of SCLC. The PES usually appears as a massive, focal, single lesion that has non-specific enhancement in CT (13) and does not have any specific characteristics that can be recognized. Therefore, PES was not initially suspected by imaging.

The preoperative tumor markers in Case 1 were a ProGRP of 253.60 pg/mL and in Case 2 a ProGRP of 150.13 pg/mL and NSE of 26.80 ng/mL. Based on clinical practice and recommendations, these values are indicative of SCLC. Although no particular marker has been identified in Ewing sarcoma family of tumors (ESFT), Lawlor et al. determined that EWS is frequently associated with the expression of the GRP gene (14). Literature reports that ProGRP levels are directly related to the size of the tumor and are similar to the ProGRP immunoreactivity of EWS cells. Thus, ProGRP can be used as a marker to diagnose and monitor ESFT but is less sensitive in EWS (about 50%) than in SCLC (15). As has already been shown by other studies, ProGRP has a strong specificity to identify SCLC as one of its major tumor markers (16). When it comes to SCLC, a ProGRP measurement greater than 200 pg/mL will give a diagnostic confidence of more than 90 percent even with abnormal renal function (17). Alternatively, the values of ProGRP in PES usually remain within physiological ranges, although sometimes a slight increase may occur. Both patients in the two cases in this report had normal renal function, but in the first case there was a ProGRP level as high as 253.60 pg/mL which was a strong indicator of SCLC. This led to diagnostic confusion in clinical practice. However, this observation also suggests that ProGRP is not the unique tumor marker of SCLC, since ProGRP values in PES patients can also be very high, which complicates the process of distinguishing between the two. Finally, the differential diagnosis should be based on molecular pathology tests, including immunohistochemistry or EWSR1 gene rearrangement.

EWS is histologically characterized by tightly packed small round blue cells, which are similar to SCLC, synovial sarcoma, neuroblastoma, and lymphoblastic lymphoma, making them hard to differentiate (18). Squamous cell carcinoma had been ruled out in Case 1 on the basis of morphology but SCLC was still a major differential. Moreover, this pathology pitfall led to a possible consideration of SCLC in Case 2 when there was an examination of intraoperative frozen sections and biopsies.

CD99, NKX2.2 and FLI-1 are core IHC diagnostic markers for PES. CD99 (a product of the MIC2 gene) is typically expressed diffusely and strongly on the membranes and may be used as a reliable EWS marker (19), although it is not specific. FLI-1 is almost specific at 88 percent (20) and NKX2.2 is highly specific. The specificity of the CD99/NKX2.2 dual-positive expression pattern is reported to be 98 percent of EWS (21). The simultaneous positivity of CD99, NKX2.2, and FLI-1 also adds to the diagnostic specificity. However, the molecular hallmark of SCLC is the presence of RB1 and TP53 gene mutations, which cause the functional inactivation of RB1 and the abnormal upregulation of p53 protein (22). Immunohistochemical (IHC) findings of Case 1 of this report revealed positivity of CD99 (+), FLI-1 (+), and NKX2.2 (+) (**Figures 4D–F**), and sporadic positivity of S-100 (scattered +), partial positivity of CK (AE1/AE3), (partial +), and focal positivity of TLE1 (focal +) (**Figures 4G–I**). With the positive EWSR1 gene rearrangement indicating gene breakage, combined with the clinical examination, a definitive diagnosis of PES was made that effectively excluded SCLC. In Case 2, the double-positivity of CD99 and NKX2.2 was sufficient to confirm the diagnosis of PES.

It is crucial to acknowledge that besides the traditional diffuse and intense positivity of CD99, the immunophenotype of Case 1 also showed scattered positivity of S-100 (scattered +) (**Figure 4G**), an uncommon finding in PES. Few reports of osseous and soft tissue EWS have noted the same S-100 (+) that probably represents a neurogenic differentiation lineage within the tumor mass. S-100 (+) is frequently found in neurogenic tumors and may also be present in lymphoblastic lymphoma and synovial sarcoma. Due to their similarities in histology with EWS, differential diagnosis is necessary (5). Moreover, the IHC findings of Case 1 in the present report also showed partial positivity rates of cytokeratin CK (AE1/AE3) (partial +) and focal positivity rates of TLE1 (focal +) (**Figures 4H, I**). Although synovial sarcoma usually displays immunoreactivity to CK (AE1/AE3) or epithelial membrane antigen (EMA) and TLE1 (23), the poorly differentiated small cell variety can demonstrate only focal weak CK staining and possible immunoreactivity to membranous CD99 and S-100 (24). It is extremely close to the partial IHC findings of the EWS in Case 1 and can easily be mistaken for poorly differentiated synovial sarcoma. Moreover, in all PES instances documented so far, it is uncommon to find either S-100 or CK positivity, and no instance has been reported with a combination of partial positivity of S-100, CK and TLE1. It also makes differential diagnosis with EWS even more challenging. The above-listed differential diagnoses clearly indicate the importance of using molecular genetic testing in case of confusing IHC expression.

The t (11,22)(q24;q12) translocation drives EWSR1 disruption and subsequent EWS-FLI1 fusion, defining features of the Ewing sarcoma family of tumors (6). Consequently, a definitive diagnosis of EWS can be made by conducting molecular tests like FISH or RT-PCR. Molecular testing done in Case 1 of this report showed that the EWSR1 gene was positive and broken, which confirmed that it was PES. Nevertheless, in Case 2, due to economic constraints (though the definitive IHC pattern was highly suggestive of PES), additional molecular testing could not be done, and the ultimate diagnosis was also found to be PES.

The clinical studies on PES are still limited, and a comprehensive treatment regimen has not been developed. Currently, treatment primarily involves radical surgery followed by systemic adjuvant chemotherapy (25). Patients with contained pulmonary PES can achieve a 5-year disease-free survival (DFS) probability of 60–70 percent (26). The conventional VDC/IE regimen is the most widely recognized among all chemotherapy protocols (27). Postoperative radiotherapy may be considered for patients with incomplete tumor resection, though its therapeutic efficacy remains unclear (28). In Case 1, the patient received post-surgical alternating VAC/IE chemotherapy and showed no evidence of recurrence at the latest follow-up. In contrast, Case 2 was lost to follow-up and did not receive adjuvant chemotherapy. All patients above were misdiagnosed as SCLC during the diagnostic process. Although both cases were initially misdiagnosed as SCLC and finally confirmed as PES, surgical resection ensured unimpeded treatment. This also serves as a reminder that in the case of such patients, where SCLC is hard to rule out, maximum efforts need to be made to acquire adequate tissue by preoperative biopsy. When clinical features do not correspond with the characteristic clinical features of the pathological subtype under consideration of SCLC, EWSR1 molecular testing should be done as part of a comprehensive pathological assessment, as far as it is feasible. When pathological evaluation cannot be performed for different reasons and SCLC remains unexcluded, whether initial surgical resection can be performed so that no barriers to treatment progress occur and thus the chances of patient survival can be increased is an issue worth investigating.

## 4 Conclusion

This study reported two cases of non-smoking female patients admitted for hemoptysis and back pain as the chief symptoms. Although hemoptysis and back pain were the main clinical symptoms, both were ultimately diagnosed with primary PES. PES lacks specific clinical manifestations. In this study, both patients presented with pulmonary masses combined with hemoptysis or back pain, which are typical manifestations of primary lung malignancies and easily lead to misdiagnosis. Thus, the presence of PES cannot be dismissed in young or middle-aged adults lacking established lung cancer risk factors who present with hemoptysis or in older adults with back pain, even when the latter is an established sign of lung cancer diagnostics. PES should be added to the differential diagnoses for patients with elevated ProGRP levels and histological small round-cell tumors, as such findings are conventionally considered specific indicators of SCLC. When there are positive results of CD99 (+), FLI-1 (+), and NKX2.2 (+) and also scattered S-100 (+), partial CK (AE1/AE3) (+), and focal TLE1 (+) on immunohistochemistry, it is important to rule out PES, SCLC, and synovial sarcoma; in these particular settings, molecular pathological testing serves as the gold standard for definitive diagnosis. In situations where exclusion of SCLC is challenging, all reasonable attempts should be taken to conduct a full-scale pathological assessment, such as EWSR1 molecular analysis. Moreover, surgical resection can be considered to avoid delayed optimal treatment.

## Data Availability

The original contributions presented in the study are included in the article/supplementary material. Further inquiries can be directed to the corresponding author.
